# Physiological Responses and Gene Expression Patterns in Open-Pollinated Seedlings of a Pummelo-Mandarin Hybrid Rootstock Exposed to Salt Stress and Huanglongbing

**DOI:** 10.3390/plants10071439

**Published:** 2021-07-14

**Authors:** Lamiaa M. Mahmoud, Patrick J. Huyck, Christopher I. Vincent, Frederick G. Gmitter, Jude W. Grosser, Manjul Dutt

**Affiliations:** 1 Citrus Research and Education Center, University of Florida, Lake Alfred, FL 33850, USA; lamiaa.mahmoud@ufl.edu (L.M.M.); Patrick.Huyck@uga.edu (P.J.H.); civince@ufl.edu (C.I.V.); fgmitter@ufl.edu (F.G.G.J.); jgrosser@ufl.edu (J.W.G.); 2 Pomology Department, Faculty of Agriculture, Mansoura University, Mansoura 35516, Egypt

**Keywords:** *Candidatus* Liberibacter asiaticus (*Ca*Las), salt stress, citrus rootstocks, genetic improvement

## Abstract

Huanglongbing (HLB), caused by the phloem-limited bacterium *Candidatus* Liberibacter asiaticus (*Ca*Las), is the primary biotic stress causing significant economic damage to the global citrus industry. Among the abiotic stresses, salinity affects citrus production worldwide, especially in arid and coastal regions. In this study, we evaluated open-pollinated seedlings of the S10 (a diploid rootstock produced from a cross between two siblings of the Hirado Buntan Pink pummelo (*Citrus maxima* (Burm.) Merr.) with the Shekwasha mandarin (*Citrus reticulata* Blanco)) for their ability to tolerate HLB and salinity stresses. In a greenhouse study, ‘Valencia’ sweet orange (either HLB-positive or negative) was grafted onto six clonally propagated lines generated from the screened seedlings in the greenhouse and the trees were irrigated with 150 mM NaCl after eight months of successful grafting and detection of *Ca*Las in the leaf petioles. Cleopatra mandarin was used as a salt-tolerant and HLB-sensitive rootstock control. *Ca*Las infection was monitored using a quantitative polymerase chain reaction before and after NaCl treatments. Following three months of NaCl treatment, ‘Valencia’ leaves on the S10 rootstock seedlings recorded lower levels of chlorophyll content compared to Cleopatra under similar conditions. Malondialdehyde content was higher in HLB-infected ‘Valencia’ grafted onto Cleopatra than in the S10 lines. Several plant defense-related genes were significantly upregulated in the S10 lines. Antioxidant and Na^+^ co-transporter genes were differentially regulated in these lines. Based on our results, selected S10 lines have potential as salt-tolerant rootstocks of ‘Valencia’ sweet orange under endemic HLB conditions. However, it is necessary to propagate selected lines through tissue culture or cuttings because of the high percentage of zygotic seedlings derived from S10.

## 1. Introduction

Citrus is a major horticultural crop grown throughout the sub-tropics. In recent years, Huanglongbing (HLB), caused by *Candidatus* Liberibacter asiaticus (*Ca*Las), has emerged as a destructive disease that affects all commercial citrus varieties, and it is currently threatening the existence of Florida’s citrus industry [1]. In addition to the plethora of other biotic stresses affecting citrus, such as citrus canker (*Xanthomonas citri* subsp. Citri), citrus black spot (*Guignardia citricarpa*), citrus tristeza disease (CTV), and citrus phytophthora disease [2], citrus plants are also affected by abiotic stresses such as salinity and extreme temperature fluctuations [3] Climate change also influence sea-level rise, which could threaten citrus production and sustainability, especially in coastal regions [4]. Most citrus cultivars are highly sensitive to salt [5], and the commonly grown ‘Valencia’ sweet orange (*Citrus* × *sinensis* (L.) Osbeck) had decreased rind thickness, delayed maturation, and reduced yields when grown in a saline environment [5,6,7,8,9]. Thus, developing new rootstock and scion varieties, either through conventional breeding or genetic transformation, that possess superior abiotic and biotic stress tolerance-specific traits is imperative for maintaining sustainable citriculture [10,11,12,13,14].

Scions that have been budded to rootstocks are utilized for commercial citrus cultivation, and proper rootstock selection plays an important role in tree health, fruit quality, and production [15]. Additionally, rootstocks are selected to confer enhanced abiotic or biotic stress tolerance to the aboveground scion [16]. In Florida, most of the major rootstocks, such as Cleopatra mandarin (*Citrus reshni* hort. ex Tanaka), Swingle citrumelo trifoliate hybrid, [*Citrus* × *paradisi* Macfad. × *Poncirus trifoliata* (L.) Raf.], and sour orange (*Citrus* × *aurantium* L.) perform poorly under HLB pressure with 30–50% root mass loss following infection [17]. This results in decreased nutrient and water uptake, which can affect the health of the scion [18]. Thus, rootstocks are important in HLB disease management, especially in Florida, where citrus growers must produce crops under endemic HLB conditions [19,20].

Several rootstocks, both diploid and tetraploid, have been produced by the University of Florida’s citrus breeding program [21,22,23,24]. The S10 rootstock is a diploid rootstock produced from a cross between two siblings of the Hirado Buntan Pink pummelo (*Citrus maxima* (Burm.) Merr.) with the Shekwasha mandarin (*C. reticulata* Blanco). It was selected through a greenhouse salinity screening assay. Cleopatra mandarin is the leading salt-tolerant citrus rootstock in Florida [25]. However, *Ca*Las graft-inoculated seedlings of Cleopatra showed sensitivity to HLB disease [26]. We observed the S10 seed parent to be a vigorous HLB-tolerant tree with fewer visual symptoms and little damage to fruit yield/quality. Additionally, we have previously observed seedling populations from two S10 siblings to be salt tolerant [13].

Plants have acquired various stress tolerance mechanisms including physiological and biochemical changes to adapt to osmotic stress. Polyphenol’s accumulations assist plants to acclimatize to unfavorable conditions [27,28]. The phenylalanine pathway is an important process in the plant cell to defend against biotic and abiotic stress. During the biosynthesis of phenolic compounds, erythrose 4-phosphate is combined with phosphoenolpyruvate (PEP) to form phenylalanine. Then phenylalanine ammonia lyase (PAL) plays an essential role to catalyzes the conversion of phenylalanine to trans-cinnamic acid, then benzoic acid. A set of phenolic compounds such as flavonoids, salicylic acid, coumarins, tannins, lignans, lignins, monolignols, and hydrolysable is formed through this pathway. [29,30,31]. Conversion of trans-cinnamic to benzoic acid leads to salicylic acid synthesis, which is an important phytohormone against biotic and abiotic stress. Many transcription factors respond to the biotic and abiotic stresses to reduce ion homeostasis in the cell such as ROS-scavenging enzymes [32], pathogenesis-related proteins (PRs) [33,34], and ion transporter factors [35,36]. ROS-scavenging enzymes are important to detoxify O^2−^ and H_2_O_2_ and decrease the toxic effect. Superoxide dismutases (SOD) play a leading role in the plant defense mechanism as they rapidly convert O^−2^ to H_2_O_2_. The PR group of proteins is commonly known as pathogenesis-related proteins [37] and is usually activated during biotic stress [38]. However, there is evidence of the crosstalk in response to abiotic and biotic stresses in plants [39,40]. The expression of most of the PR genes remains at the basal level under normal conditions. Their expression increases dramatically under stress. In Arabidopsis, some PR genes (*PR1*, *PR2,* and *PR5*) were upregulated by abiotic stress [34,41]. The role of ion transporters in the plant cell is (1) to control the entry and exit of Na^+^ into and out of plant cells, (2) to regulate Na^+^ compartmentation in the vacuoles, and 3) to selectively import K^+^/Na^+^ into plant cells [42]. The salt overly sensitive (SOS) pathway was identified through a salt study of Arabidopsis mutants [43]. Studying the response of *sos1*, *sos2*, and *sos3* mutants to salt showed a principal role of ion homeostasis in maintaining the cytoplasm in salt stress tolerance [42,44]. Na^+^/H^+^-antiporter (*NHX*) also has an effective function in controlling the movement and transport of Na^+^ or K^+^ ions into the vacuoles in exchange for H^+^ efflux to the cytosol [36].

**Figure 1:**
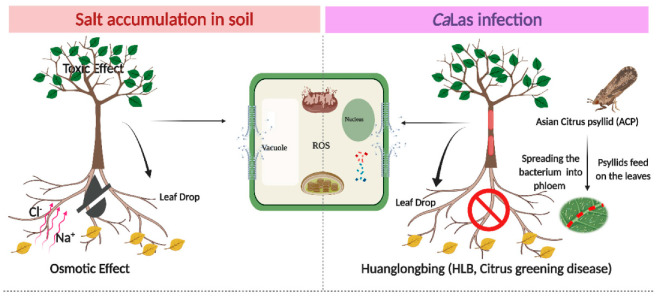
Schematic diagram of the interactive effect of abiotic stress (salt stress) and biotic stress (HLB) in the plant cell. The left photo shows the effect of sodium and chloride accumulation in soil, and the negative effects of NaCl stress resulting from three main factors: (1) The osmotic effect of increasing salt ions in the soil or apoplast, which decreases the availability of external water and creates a more negative internal tissue water potential as salt is taken up, (2) the toxicity of specific ions, and (3) the nutritional imbalance. On the right, HLB disease occurs following the movement of *Ca*Las through the phloem tissues. This results in blockage in the phloem tissues and impaired nutrient transport. Subsequently, the plant cells produce toxic reactive oxygen species (ROS) in the two molecular forms (free radicals and non-radical). ROS overproduction results in DNA and protein damage, lipid peroxidation, impairment of cell metabolism, interruption of cellular homeostasis, and consequently, cell death. The symptoms appear as leaf necrosis, growth suppression, leaf abscission, a decline in fruit production, and finally, plant death. The figure was created in BioRender.com.

The primary salt present in seawater is NaCl (2.3%) with calcium, magnesium, potassium, and other salts making up the remainder of ions [45]. Because of the sensitivity of citrus trees to both salinity stress and HLB (Figure 1), the identification of tolerant rootstocks that may mitigate HLB damage and thrive in saline soils is important. In the current study, we evaluated the ability of open-pollinated S10 lines to grow in NaCl-stressed soil, following *Ca*Las infection in relation to Cleopatra mandarin. In order to screen for ‘Valencia’ scions to be grafted on the best clone in the environment NaCl + *Ca*Las infectionin, we focused on biochemical markers such as the content of total chlorophyll, starch, total phenolic compounds (TPC), proline, malondialdehyde (MDA), and sodium and chloride ions. Additionally, we studied the relative expression of superoxide dismutases (SOD) and phenylalanine ammonia lyase (PAL) enzymes, two of PR genes (*PR1* and *PR2*) and ion transporters (*SOS1*, *SOS2*, *SOS3,* and *NHX1*).

## 2. Results and Discussion

### 2.1. Simple Sequence Repeat (SSR) Marker Analysis

Two hundred S10 seedlings were germinated and screened for salt tolerance in the greenhouse through the exogenous application of 150 mM NaCl. Under constant NaCl stress, most seedlings did not survive. We identified 15 seedlings that grew well under the constant 150 mM NaCl stress. All these seedlings were clonally propagated, and six random lines with an adequate number of cuttings necessary for the physiological experiments were selected. First, we conducted molecular analyses on these six lines using six EST-SSR markers to confirm their genetic origin. The degree of genetic similarity among the seedlings was determined and compared with that of the hybrid parents. Analysis of the EST-SSR molecular markers indicated that five seedlings were zygotic open pollinated seedlings of unknown pollen parentage while the sixth was nucellar and like the seed parent (Table 1).

Nucellar seedlings arise from maternal tissues and are genetically identical to the maternal genotype [46]. Most commercially available rootstock cultivars produce seeds that contain mostly nucellar embryos. Zygotic seedlings that contain the genetic information of both pollen and seed parent are produced by some commercial rootstocks [47]; however, they are generally eliminated by alert nursery workers, although it can sometimes be difficult to identify them during the early stages [48]. Zygotic rootstocks can, however, be propagated through tissue culture, which allows it to maintain the genetic fidelity of the cultivar [49].

### 2.2. Effect of NaCl Treatment and CaLas on Total Chlorophyll and Starch Accumulation in Leaves

The nucellar seedling S10-6 (S10-control) obtained in this study, being genetically identical to the seed parent, was utilized as a control. This line was compared against the other five zygotic lines and the industry-standard Cleopatra salt-tolerant mandarin. Physiological parameters of ‘Valencia’ sweet orange budded onto the different rootstocks were evaluated. Significant variance analysis among rootstocks, NaCl stress, and *Ca*Las infection was implemented using a three-way ANOVA. Our analysis revealed that the rootstock, NaCl treatments, and *Ca*Las infection significantly affected the physiological variables (Supplementary Table S1). The total chlorophyll content was significantly decreased by the interaction effect of NaCl treatment and *Ca*Las infection in ‘Valencia’ grafted onto Cleopatra (3.01 mg^−1^ g FW), and the total pigment content ranged between 3.73 and 5.90 mg^−1^ g FW in the S10 lines (Table 2). There were minor differences in T Chl in ‘Valencia’ leaves grafted onto S10 rootstocks compared to the T Chl in ‘Valencia’ grafted onto Cleopatra under NaCl treatment without *Ca*Las infection. *Ca*Las infection significantly increased foliar starch content that doubled when the infected plants were treated with NaCl. The *Ca*Las-infected ‘Valencia’ grafted onto Cleo, S10-control, S10 lines 3 and 5 accumulated the highest levels of starch in the leaves following exogenous application of 150 mM NaCl (Table 2). 

Excessive accumulation of starch granules in photosynthetic cells, vascular parenchyma, and phloem elements can be used as a reliable marker for HLB infection in citrus [50]. Starch accumulation in the infected plants can result in phloem blockage so that normal sugar export from the leaf is partially limited, and thus photosynthate accumulates as starch. Such accumulation in leaves has also been found in response to salt stress in some organisms, though it is contrary to general stress responses where sugar accumulates as non-toxic osmotic solutes, and starch is deleted in leaves, thus serving as a source of carbohydrate when stress has inhibited photosynthesis [51]. Thalmann [51] and Dong [52] review several mechanisms that have been suggested to explain situations where there is an accumulation of starch under salt stress. These mechanisms include: (1) salinity might induce accumulation of sugar as an osmotic response, but the response may be excessive, and the conversion of some sugar to starch might help protect the leaf from damage [52]; (2) sink-organ growth might be inhibited by salt stress such that there is limited export of carbohydrate to the sinks, which in turn leads to a backlog of sugar at the source while there is some continued photosynthesis, and the leaf responds by partitioning more carbohydrate into leaf starch [53]; and (3) recent evidence in *Phragmites australis* (common reed) suggests that starch may “flocculate” or scavenge the toxic ions and alleviate their systemic spread to sensitive tissues [54]. Given the present results, *Citrus* appears to fall within the group in which foliar starch accumulation occurs under saline conditions, and this effect is exacerbated by *Ca*Las infection.

### 2.3. Effect of NaCl Treatment and CaLas on TPC, Proline and MDA Content

Total phenolic compounds (TPC) content was determined in ‘Valencia’ leaves to evaluate the NaCl-induced oxidation response in the rootstocks. TPC content in the lines S10-line 3, 4, 5, and S10-control were similar to Cleopatra in response to NaCl treatment (Table 3). *Ca*Las-infected ‘Valencia’ had increased TPC content when exposed to NaCl treatment with the highest TPC (90.33 mg g^−1^ FW GAE) in the leaves of ‘Valencia’ grafted onto S10-line 6, followed by ‘Valencia’ grafted onto Cleopatra and S10-line 5 (80.33, 74.67 mg gallic acid g^−1^ FW; Table 3). ‘Valencia’ grafted onto S10-3 recorded the highest proline content when they were irrigated with NaCl (221.33 µmol g^−1^ FW), followed by ‘Valencia’ grafted onto S10-line 6 and S10-line 1 (202.67 and 201.13 µmol g^−1^ FW), respectively. In the *Ca*Las-infected ‘Valencia’ group, trees budded onto S10-line 1 recorded the highest proline following NaCl treatment (227.00 µmol g^−1^ FW). Healthy ‘Valencia’ grafted onto Cleopatra recorded the lowest proline content in both conditions. Lipid peroxidation in the leaves of ‘Valencia’ and roots of the S10 seedlings rootstocks were measured as MDA content (Table 3). The MDA content increased under NaCl treatment; healthy ‘Valencia’ grafted onto S10-line 2 leaves had less damage (38.21 nmol^−1^ MDA eq. g FW) amongst the evaluated rootstocks. The lowest MDA content in the leaves in the *Ca*Las-infected lines that had been treated with NaCl was observed in ‘Valencia’ grafted onto S10- lines 4, 3, and 2, (76.51, 84.24, and 86.71 nmol^−1^ MDA eq. g FW), respectively. MDA levels were higher for *Ca*Las-infected trees than healthy ones. The lowest content of MDA in roots was recorded in S10- lines 3, 5, 2, Cleo, and 1, respectively, under NaCl treatment. MDA content increased in most of the infected rootstocks and those treated with NaCl except S10- line 1 and S10- line 2. These lines also had less damage in the roots (11.57 and 11.17 nmol^−1^ MDA eq. g FW, respectively) following NaCl treatment and *Ca*Las infection, followed by S10-line 3 (20.49 nmol^−1^ MDA eq. g FW). The highest MDA content in the roots was recorded in the S10-line 5 and Cleopatra (34.49 and 33.89 nmol^−1^ MDA eq. g FW) followed by S10-line 4 and S10-line 6 (30.89 nmol^−1^ MDA eq. g FW for both).

The interaction effect between biotic and abiotic stresses is complex. In many cases, induced abiotic stress on a plant can either decrease or increase the ability of the plant to combat biotic stress [55]. An adverse effect of NaCl ion accumulation in the soil increases the osmosis level in the soil, subsequently lowering the available water in the soil [56]. In addition, the osmotic effect that occurs due to salinity levels causes toxic effects and nutritional imbalance [13]. In general, *Ca*Las causes the blockage of phloem tissues, which leads to difficulty in nutrient absorbance [57]. Thus, the interaction of NaCl treatment with *Ca*Las results in the overproduction of reactive oxygen species in the plant cells and potentially causes damage to the plant cell [58]. In general, the exogenous NaCl stress application resulted in an increase in MDA content in the citrus rootstocks. NaCl stress leads to the overproduction of free oxygen radicals that can potentially break the cell membrane and induce membranous lipid peroxidation [59]. The final product of this process is MDA, which is a marker for cell membrane damage [60]. 

### 2.4. Effect of NaCl Treatment and CaLas Infection on Sodium and Chloride Content in Leaves and Roots 

Under the NaCl treatment, ‘Valencia’ trees grafted onto S10-line 2 had the highest Na^+^ content in the leaves (Table 4). In contrast, ‘Valencia’ grafted onto S10- line 6 retained significantly lower Na^+^ content in the leaves (Table 4). ‘Valencia’ grafted onto S10- lines 1, 4, 5, 6, and Cleo also showed significantly higher Na^+^ content in the leaves, whereas S10- lines 1, 2, 3, and 4 recorded high levels of Na^+^ in the roots of the infected rootstocks following NaCl treatment (Table 4). The Cl^−^ content in the roots of all S10-lines was statistically similar to that in Cleopatra roots in saline conditions. In the *Ca*Las-infected trees supplemented with exogenous NaCl treatment, S10 lines showed similar performance of Cl^−^ inclusion in the roots as they performed without *Ca*Las infection, but ‘Valencia’ grafted onto S10- lines 3 and 4 showed lower content of Cl^−^ in the roots compared to the controls. 

Sensitivity to salt stress is mainly due to chloride toxicity [61]. Salt-tolerant citrus rootstocks accumulate copious amounts of Na^+^ and Cl^−^ in their roots and decrease transport to the aerial parts to avoid oxidative damage in chloroplasts and the subsequent impairment of photosynthesis [62]. Most S10 lines recorded a similar response in chloride accumulation in the roots as the salt-tolerant Cleopatra, which suggests that S10 is as tolerant to salt stress as the control. We observed a change in the performance of the infected Cleopatra following salt stress and after *Ca*Las infection. This difference may be due to the sensitivity of Cleopatra to *Ca*Las [63]. HLB is known to reduce the performance of the fibrous root [64] and we could correlate the reduction in root function with the high levels of MDA since it indicates the high damage in the roots. This indicated the cellular damage in ‘Valencia’ leaves and roots on Cleopatra. HLB also causes blockage of the phloem tissues in the infected plants [50] and may result in changes to Na^+^ and Cl^−^ ions movement in the HLB susceptible Cleopatra. The low content of MDA in the roots of S10 line 2 indicates the integrity of the roots in these rootstocks following HLB infection. This observation, along with the insignificant differences in Cl^−^ content in both leaves and roots in S10-2, ascertains the tolerance to salt and HLB stresses of S10. Therefore, the S10- line 2 performed similar or higher than Cleopatra rootstock under NaCl treatment and S10- line 2 was superior to Cleopatra rootstock under NaCl treatment and *Ca*Las infection. 

### 2.5. CaLas Diagnosis and Gene Expression Analysis

To investigate the presence of *Ca*Las in the ‘Valencia’ scions, we performed qPCR analysis using genomic DNA isolated from the leaf petioles. Bacteria were detected before and after the NaCl treatment cycle, indicating the stability of the *Ca*Las inside the ‘Valencia’ phloem (Table 5). The Ct values in ‘Valencia’/S10 rootstock ranged from 25.30 to 31.45 after eight months of inoculation and before NaCl treatments, whereas it was 26.87 in the Cleopatra control. In all lines, the Ct values decreased following the NaCl stress treatment, although the differences were only significant in the Cleopatra control. The Ct values in the S10 seedlings did not decrease at the same rate as that observed with the Cleopatra mandarin control, which is a *Ca*Las sensitive cultivar [58] indicating that S10 can better withstand *Ca*Las than Cleopatra, even under NaCl treatment conditions.

### 2.6. Gene Expression in the Citrus Scion and Rootstocks

**Figure 2:**
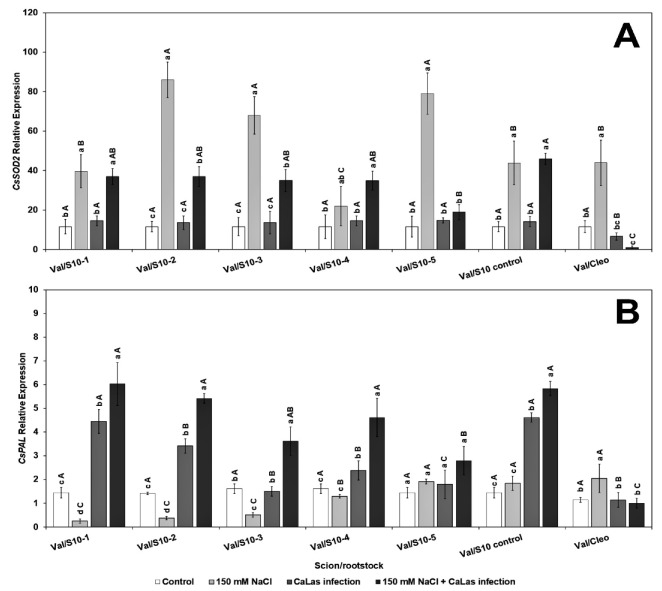
Effect of NaCl stress and *Ca*Las infection on ‘Valencia’ sweet orange scions grafted onto S10-derived rootstocks and Cleopatra mandarin (control). (**A**), *CsSOD2*, (**B**), *CsPAL*. Bars represent means ± standard error. Mean separation between rootstocks at a particular treatment is indicated by differing uppercase letters; mean separation between treatments in a particular rootstock is indicated by differing lowercase letters by Tukey’s honestly significant difference test (*p* ≤ 0.05).

Genes that play a major role in the abiotic and biotic stress pathways were chosen for evaluation in the present study. The transcript level of *CsSOD2* (chloroplastic CuZnSOD, superoxide dismutase), which contributes to reducing oxidative stress [65], significantly increased in response to NaCl treatment in all ‘Valencia’ lines scions grafted onto the S10 lines compared to *Ca*Las infected Val/Cleo under NaCl treatment (Figure 2A). *CsSOD2* activity was minimal in the *Ca*Las-infected Val grafted onto Cleo that had been treated with NaCl. Superoxide dismutase plays an effective role in plant defenses as they rapidly convert O^−2^ to H_2_O_2_ [66]. 

However, the level of *CsPAL* (phenylalanine ammonia lyase), which is also upregulated by abiotic stresses [67] was only highly upregulated in the infected ‘Valencia’ grafted onto S10-line 1, 2, and S10-line 6 (S10-control) (Figure 2B). The other lines (except S10- line 3) were all significantly different from Val grafted onto Cleo, both after NaCl-induced stress and the NaCl–*Ca*Las combined conditions. PAL is the most important enzyme controlling the phenylpropanoid pathway, and it aids in producing several metabolites that regulate various environmental stresses [68]. Thus, with increasing the expression of this gene in S10- line 1 and S10-line, five of our experimental rootstock lines provides evidence of the increased accumulation of beneficial phenolic protectant compounds to alleviate both *Ca*Las- and NaCl-induced stresses.

**Figure 3:**
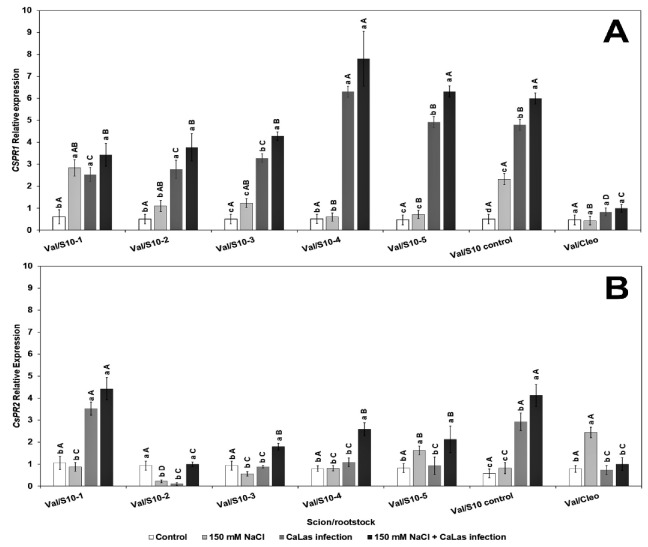
Effect of NaCl stress and *Ca*Las infection expression of defense-related genes in ‘Valencia’ sweet orange scions grafted onto S10-derived rootstocks or Cleopatra mandarin (control). (**A**), *CsPR1* and (**B**). *CsPR2*. Mean separation between rootstocks at a particular treatment is indicated by differing uppercase letters; mean separation between treatments in a particular rootstock is indicated by differing lowercase letters by Tukey’s honestly significant difference test (*p* ≤ 0.05).

The relative expression levels of two PR genes, *CsPR1* and *CsPR2,* were evaluated in the present study. PR1 and PR2 are induced following *Ca*Las infection [33,69,70]. Our results suggested that there was a significant difference in the two tested genes in most of the experimental S10 lines (Figure 3). *CsPR1* gene expression upregulated in response to *Ca*Las infection with or without NaCl treatment had the highest PR1 gene expression in S10- lines compared to Val/Cleo (Figure 3A). PR1 was upregulated in line S10- line 1 and S10- line 6 following NaCl treatment. PR2 was highly upregulated following *Ca*Las infection and NaCl treatment in Val/S10- line 1 and Val/S10-control upregulated in response to *Ca*Las infection with or without NaCl treatment (Figure 3B). Overall, in some of the lines, the PR genes were upregulated following *Ca*Las infection coupled with NaCl treatment. Thus, the PR genes that are usually induced following biotic stress can also be induced in tandem with abiotic stress. While the S10-control line (nucellar offspring of the parent tree) performed well, most of the zygotic seedlings demonstrated better PR gene induction. PR genes are significant key components of the plant’s innate immune system especially systemic acquired resistance (SAR) to enhance plant defense against abiotic and biotic stress and are widely used as molecular markers of defense signaling pathways [37]. After pathogen attack, plants activate defense signaling pathways involving salicylic acid (SA) and jasmonic acid (JA), which further enhance PR proteins accumulation that defend and minimize pathogen load or disease stress. SA pathway stimulates the transcription of NPR1 (non-expressor of pathogen-related gene 1) that activates SA signature genes (PR1, PR2, and PR5) products locally as well as systematically, leading to systemic acquired resistance (SAR) [37]. Although, the effect of abiotic stress on the expression of PR genes is not fully understood at the molecular level, some previous reports have shown that osmotic stress significantly increases the expression of PR genes in Arabidopsis plants [34,41]. Additionally, mRNA levels of PR1 in pepper plants significantly increased during abiotic stresses [71]. When the plants grow under a combined abiotic and biotic challenge, they may conduct complex pathways to simulate SAR genes to increase plant tolerance.

**Figure 4:**
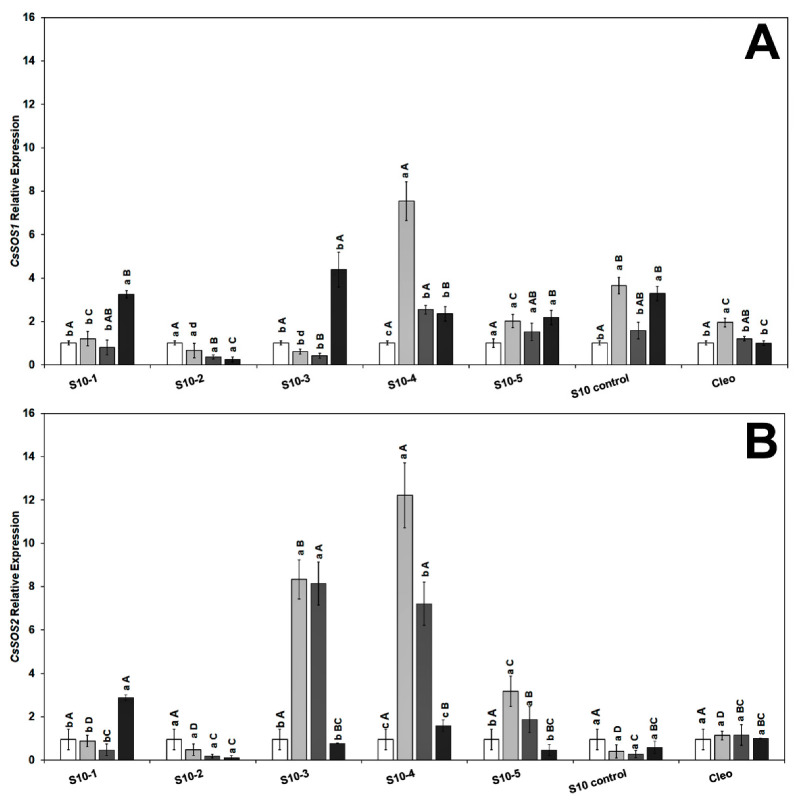
Effect of NaCl treatment and *Ca*Las infection on ‘Valencia’ sweet orange scions grafted onto S10-derived rootstocks and Cleopatra mandarin (control). (**A**) *CsSOS1* and (**B**) *CsSOS2*. Data represent means ± standard error. Mean separation between rootstocks at a particular treatment is indicated by differing uppercase letters; mean separation between treatments in a particular rootstock is indicated by differing lowercase letters by Tukey’s honestly significant difference test (*p* ≤ 0.05).

To gain further insight into the molecular mechanisms underlying the role of NaCl tolerance, we investigated the expression level of the salt overly sensitive genes (SOS), *CsSOS1*, *CsSOS2*, and *CsSOS3*, and the Na^+^/H^+^ exchanger *CsNHX1* under NaCl treatment and *Ca*Las infection in the feeder roots because these genes have been implicated as playing a key role during the salinity tolerance process [43,72]. We did not observe any specific trend in either the SOS or Na^+^ transporter genes, and the genes were overexpressed following NaCl treatment alone or combined with *Ca*Las infection. The transcript levels of *CsSOS1* (salt overly sensitive 1) were upregulated in response to *Ca*Las infection in all lines (Figure 4A). The *CsSOS2* (salt overly sensitive 2) gene was highly upregulated in the S10-3 and S10-4 roots treated with NaCl. Expression levels decreased following infection with *Ca*Las in all S10-lines except S10 line -1 compared to infected Cleo under NaCl treatment (Figure 4B). 

**Figure 5:**
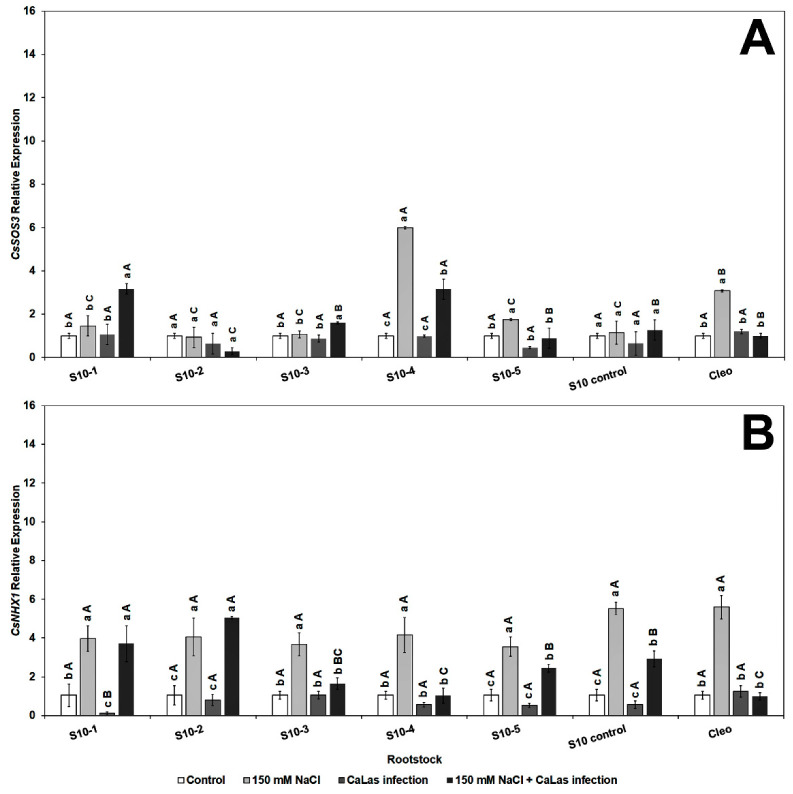
Effect of NaCl treatment and *Ca*Las infection on ‘Valencia’ sweet orange scions grafted onto S10-derived rootstocks and Cleopatra mandarin (control). (**A**) *CsSOS3* and (**B**) *CsNHX1*. Data represent means ± standard error. Mean separation between rootstocks at a particular treatment is indicated by differing uppercase letters; mean separation between treatments in a particular rootstock is indicated by differing lowercase letters by Tukey’s honestly significant difference test (*p* ≤ 0.05).

The *CsSOS3* (salt overly sensitive 3) gene was significantly overexpressed in response to *Ca*Las infection and NaCl treatment in S10-1, 3, 4, 5, and S10-control (Figure 5A). None of the SOS genes were highly upregulated in the S10- line 2; however, there was upregulation of *CsNHX1* (Figure 5B). The *CsNHX1* (Na^+^/H^+^-antiporter 1) gene was upregulated in most lines in response to the combined stresses (*Ca*Las infection and NaCl treatment), and all the lines except S10-4 were significantly different from the infected Cleo under NaCl treatment (Figure 5B). SOS1 is overexpressed due to NaCl treatment, mainly in the epidermal cells of the root tip [73,74], and is controlled by the *SOS2* and *SOS3* regulatory pathways [75]. Apart from the S10- line 2 line, all lines overexpressed SOS1 following salt stress and *Ca*Las infection, which indicates active transport and removal of excess Na^+^ ions from the root cells. *SOS2* is upregulated in the roots and optimum expression occurs within 12 h of salt treatment [73,74]. Thus, it is possible that the lower expression levels observed in most of our lines were due to the rapid expression of this gene within the first few hours or days following NaCl treatment, and the levels might have decreased at the time of root sampling. *SOS3* is responsible for activation of SOS [76]. Thus, expression levels of *SOS2* could be correlated to *SOS3* expression at a given time. *NHX1* plays a major role in the movement of Na^+^ or K^+^ transport into the vacuoles in exchange for H^+^ efflux into the cytosol [36]. 

## 3. Materials and Methods

### 3.1. Evaluation of NaCl Tolerance of S10 Lines 

Open pollinated fruits were collected from the S10 tree, and the seeds were extracted, peeled, followed by rinsing with 1N NaOH and deionized water for 5 min. Seeds were washed twice with running deionized water to discard residual NaOH. Two hundred seeds were planted in plastic trays in a peat moss and perlite (2:1) soilless medium and kept in a 50% shaded greenhouse, under natural photoperiods. The germinating seedlings were watered with 150 mM NaCl for 60 days. The seedlings that survived the NaCl treatment were subsequently transplanted into a regular potting mix and clonally propagated in a mist-bed via single-node cuttings. Cleopatra mandarin seedings that had germinated at the same time were also clonally propagated through cuttings. When the rooted cuttings reached 15 cm in length, they were transferred to standard nursery 10 × 10 × 35 cm plastic “citripots” (standard for nursery production) containing a PRO-MIX soilless medium (Premier Tech Horticulture, Quakertown, PA, USA) and maintained in a 50% shaded greenhouse set at 30 ± 2 °C for six months. Clonally propagated trees from the six lines selected for the present study were split into two sets. The first set was cleft grafted with *Ca*Las-free ‘Valencia’ sweet orange (*Citrus* × *sinensis* (L.) Osbeck) budwood and the other set with *Ca*Las-positive ‘Valencia’ budwood. The *Ca*Las -positive budwood were collected from HLB infected trees. The presence of *Ca*Las in the scion was monitored in the source trees and the grafted rootstocks using PCR to confirm the presence of the bacteria.

### 3.2. NaCl Treatment Application and Greenhouse Conditions

The grafted trees were maintained in a greenhouse for eight months. Trees were subsequently irrigated with 500 mL NaCl solution prepared in deionized water three times per week. The NaCl concentration was increased gradually in intervals of 20 mM during the first 2 weeks, until a final concentration of 150 mM was achieved to avoid osmotic shock as outlined previously [13,32]. Similarly grafted Cleopatra mandarin cuttings were used as the salt-tolerant rootstocks, and controls were irrigated with the same deionized water without the addition of NaCl. Each treatment consisted of three replicates. 

### 3.3. Simple Sequence Repeat (SSR) Marker Analysis

To assess the genetic variation in the different S10 lines, simple sequence repeat (SSR) marker analysis on the genomic DNA was carried out. Fresh leaf samples were collected, and DNA was extracted from the leaves (100 mg fresh weight) using a GeneJET Plant Genomic DNA Purification Mini Kit (Thermo Fisher Scientific, Waltham, MA, USA) following the manufacturer’s protocol. The concentration was measured with a Nanodrop spectrophotometer (Thermo Fisher Scientific, Waltham, MA, USA) and normalized to 25 ng/µL. Six SSR primer sets (synthesized by Operon Technologies, Huntsville, AL, USA) were used in the present study. PCR amplifications were performed on a T100™ Thermal Cycler (Bio-Rad Laboratories, Inc., Hercules, CA, USA) and fragment separation was performed on an ABI PRISM 3130 xl Genetic Analyzer (Applied Biosystems, Foster City, CA, USA). A universal M13 primer (5′—GTTGTAAAACGACGGCCAGT—3′) was fluorescently labeled and added as a common tail to the 5′ end of the forward SSR primers. The generated chromatographic peaks were further analyzed with GeneMarker (SoftGenetics GeneMarker 1.40 software, State College, PA, USA) to score alleles [base pairs (bp)] for all the parents and the S10 lines and summarized in a table [22]. Alleles in each line were compared with the parents to determine whether the line is zygotic or nucellar. A nucellar plant had alleles similar to those alleles of the S10 parent. When specific alleles were not detected in the S10 lines compared to the S10 parent, we determined the line to be a zygotic seedling.

### 3.4. Physiological Variables Measurement 

Median leaves (four per plant) were collected three months after the completion of NaCl treatments. The fresh leaves (100 mg fresh weight) from all treatments were homogenized in 1 mL of absolute methanol, centrifuged at 10,000 rpm for 15 min at 4 ℃, and further diluted 10X with fresh methanol. The mixture was analyzed for chlorophyll a (Chl a) and chlorophyll b (Chl b) by observing the absorbance at different wavelengths (665 nm for chlorophyll a (Chl a) and 653 nm for chlorophyll b (Chl b)) using a spectrophotometer (Biochrom Libra UV-visible). The total chlorophyll content was calculated according to the method described by Lichtenthaler and Wellburn [77]. 

Starch quantification was performed according to Rosales and Burns [78] with a few modifications. Dried leaves (50 mg) were ground to a powder and homogenized in 700 μL of distilled water. Leaf samples and a standard (rice starch (Sigma Aldrich, St. Louis, MO, USA)) were boiled in water for 10 min. Cooled samples were subsequently vortexed and centrifuged for 2 min at 6000 rpm. Three hundred microliters of supernatant were extracted with 900 μL of absolute ethanol. The mixture was vortexed and centrifuged for 10 min at 10,000 rpm. The supernatant was discarded, and 1 mL of distilled water was added to dissolve the pellet. Fifty microliters of KI:I2 (8 mM:50 mM) were added. The quantification of starch was accomplished by monitoring color change in a spectrophotometer at 594 nm. 

Total phenolic content (TPC) was estimated according to the procedure described by Singleton and Rossi [79]. TPC was extracted in 1 mL methanol, and the methanolic extract was centrifuged at 12,000 rpm for 10 min at 20 °C. The reaction was followed by the addition of sodium carbonate (Na_2_CO_3_) at 7.5% (*w*/*v*) and incubated for 1 h at room temperature. Gallic acid was used as a standard solution in an aqueous form in the concentration range of 100 to 600 ppm. The absorbance was measured at 760 nm. The results were expressed as mg gallic acid (GAE) g^−1^ fresh weight (FW).

Proline content was estimated based on the methodology described by Bates et al. [80]. Leaves (500 mg fresh weight) were homogenized in 5 mL of aqueous sulfosalicylic acid (3% *w*/*v*) and centrifuged at 10,000 rpm for 10 min. A mixture of 2 mL of the supernatant, 2 mL of glacial acetic acid, and ninhydrin reagent (1.25 mg of ninhydrin, 30 mL of glacial acetic acid, and 20 mL of 6 M H_3_PO_4_) was incubated for 1 h at 100 °C in a water bath. The reaction was stopped by placing the test tubes in ice and subsequently vigorously mixed with 4 mL of toluene in glass tubes. After warming at 25 °C, the chromophore was measured for proline content determination at 515 nm using a UV/Vis spectrophotometer. The proline content was determined against an L-proline-derived standard curve.

Malondialdehyde (MDA) content was measured in the leaves and roots using the method described by Heath and Packer [81]. Fresh leaves were collected and extracted in 0.5 mL of 0.1% (*w*/*v*) trichloroacetic acid (TCA). The supernatant was collected by centrifugation at 14,000 rpm at 4 °C for 10 min. 2-thiobarbituric acid (TBA) at 20% diluted in TCA was added to 0.5 mL of the supernatant and incubated at 95 °C for 25 min. The tubes were incubated in ice for 10 min to stop the reaction, then evaluated for light absorption at 532 and 600 nm using a UV/Vis spectrophotometer. The amount of MDA was calculated using an extinction coefficient of 155 mm^−1^ cm^−l^. All chemicals and reagents were purchased from Sigma-Aldrich Corp. (St. Louis, MO, USA).

### 3.5. Sodium and Chloride Ion Analysis

Ten mature leaves and the whole root system were collected from each grafted tree, briefly rinsed in deionized water, and oven-dried at 60 °C for 48 h. The samples were subsequently analyzed by the dry-ashing method [82] followed by inductively coupled plasma atomic emission spectroscopy (ICP-AES) according to Munter et al. [83]. The sodium and chloride concentrations were expressed as mM L^−1^.

### 3.6. CaLas Diagnosis and Gene Expression Analysis 

DNA was extracted from fresh leaf petioles from grafted trees and extracted similar to the method used to extract SSR marker DNA. Quantitative polymerase chain reaction (qPCR) was performed with the TaqMan™ Universal PCR Master Mix (Applied Biosystems, Foster City, CA, USA) and CQUL primers [84] to amplify a fragment of the *Ca*Las rplJ/rplL ribosomal protein gene. To normalize Ct values, negative controls were obtained from leaf samples of greenhouse *Ca*Las-free ‘Valencia’ sweet orange trees that recorded “undetermined” amplification results in prior analyses, and positive controls were obtained from infected trees in the field that recorded Ct = 21.25 [85]. For gene expression analysis, ‘Valencia’ leaves and S10 seedling roots from grafted trees were collected and kept immediately in liquid nitrogen and stored at −80 °C. RNA was extracted by using a Direct-zol™ RNA Miniprep kit. cDNA was synthesized using a PrimeScript™ RT Reagent Kit (Takara Bio USA, Inc., Mountain View, CA, USA) according to the manufacturer’s protocol. The real-time PCR (qPCR) reaction mix consisted of 1 µL DNA (25 ng/µL), SYBR^®^ Green PCR Master Mix (Applied Biosystems, Foster City, CA, USA), and primers in a final volume of 20 μL according to the manufacturer’s instructions. The expression of superoxide dismutase (*CsSOD2*) [86], phenylalanine ammonia lyase (*CsPAL*) [87], two PR genes (*CsPR1* and *CsPR2*) [15], salt overly sensitive genes (*CsSOS1, CsSOS2, and CsSOS3*) [35], and the (Na^+^/H^+^-antiporter 1 (*CsNHX1*) [88] gene were studied. *CsSOD2, CsPAL, CsPR1,* and *CsPR2* genes were studied in the leaf tissues and *CsSOS1, CsSOS2, CsSOS3,* and *CsNHX1* were studied in the root tissues. Each sample was tested in triplicate and repeated twice. The infected ‘Valencia’ grafted onto Cleopatra under NaCl treatment was considered as a control for the leaf samples and the Cleopatra root in the same condition was the control for the root samples. The relative gene expression was calculated using the 2^−ΔΔCT^ method [89] as outlined earlier [32,33]. The citrus actin housekeeping gene was used as an endogenous control. The primer sequences of the genes are shown in Supplementary Table S2.

### 3.7. Experimental Design and Statistical Analysis 

The experiment was designed as a complete block randomized factorial, with three factors (rootstocks (7), *Ca*Las infection (two levels), and NaCl treatments (two levels)) with three replicates per treatment. Each replicate consisted of three plants, one tree in each container. Analysis of variance was run in R version 4.0.3 [90]. Means separations for the physiological parameters data and *Ca*Las Ct-values were run with Tukey’s honestly significant difference test (*p* ≤ 0.05). Tukey’s test was run with the JMP Pro software version 15 (SAS Institute, Cary, NC, USA).

## 4. Conclusions

Our results revealed significant differences in the physiological, biochemical, and molecular analysis following NaCl treatment or *Ca*Las infection among the different seedling-derived lines of S10 rootstock. The Ct-values of *Ca*Las-infected S10 before and after NaCl treatment indicated the ability of the S10 rootstock seedlings to tolerate infection following NaCl treatment, in contrast to the Cleopatra rootstock, where ‘Valencia’ scions budded to it were observed to be highly susceptible to HLB. A better defense mechanism, as observed in most of the S10 seedling lines, coupled with improved Na^+^ or K^+^ transport into the vacuoles, may have played an important role to increase salinity tolerance. Thus, these rootstocks (zygotic and nucellar) can be utilized as potentially salt-tolerant rootstocks of ‘Valencia’ sweet orange growing in saline soil and exposed to endemic HLB. However, since most of them were observed to be zygotic in nature and it is unclear yet whether they are mono or polyembryonic, tissue-cultured explants or cuttings can be utilized to rapidly propagate selected lines for further field tests. 

## Figures and Tables

**Table 1:** EST-SSR marker alleles [base pairs (bp)] detected in the DNA samples from open pollinated S10 lines, the S10 seed parent, and S10 hybrid’s parents.

Genotype ^a^	CX6F04	CX6F29	CX6F18	CX6F14	CX0035	CX6F18	Origin
HBP	100 ^b^	289	149/150	263/274	395	192	Parent
SHEKWASHA	106/113	269/274	150/156	274	429	197	Parent
S10	100/106	289	149/150	263/274	395/429	192/197	Parent
S10-line 1	106/113	289	150/156	274	395/429	192/197	Zygotic
S10-line 2	113	289	149	263/274	395/429	192	Zygotic
S10-line 3	106/113	269/274	149/150	263/274	395	192	Zygotic
S10-line 4	113	274/289	150	263/274	395	197	Zygotic
S10-line 5	106	289	149/150	263/274	395	192	Zygotic
**S10-line 6** **(S10-control) ^c^**	**100/106**	**289**	**149/150**	**263/274**	**395/429**	**192/197**	**Nucellar**

^a^ HBP: Hirado Buntan Pink pummelo (*Citrus maxima* (Burm.) Merr.); SHEKWASHA: Shekwasha mandarin (*Citrus reticulata* Blanco); S10: a diploid rootstock produced from a cross between two siblings of the Hirado Buntan Pink pummelo with the Shekwasha mandarin. ^b^ EST-SSR primer amplified amplicon size from chromatogram. ^c^ The line in bold (S10-line 6) was confirmed to be of nucellar origin like the S10 parent. S10-line 6 was called as S10-control in the manuscript.

**Table 2:** NaCl treatment and *Ca*Las infection on total chlorophyll and starch content of ‘Valencia’ sweet orange scions grafted onto S10-derived rootstocks and Cleopatra mandarin (control).

Variables	NaCl Treatments	*Ca*Las Infection	Scion/Rootstocks						
			Val/S10-1	Val/S10-2	Val/S10-3	Val/S10-4	Val/S10-5	Val/S10-6 (S10-Control)	Val/Cleo
T Chl (mg^−1^ g FW)	Control	Control	6.13 a B *	6.68 a B	7.23 a AB	5.98 a B	7.09 a AB	9.10 a A	7.22 a AB
	150 mM NaCl	Control	5.93 a AB	6.10 ab AB	4.99 a B	5.28 a AB	6.14 ab AB	6.78 b A	5.62 b AB
	Control	*Ca*Las infection	4.41 ab B	5.60 ab AB	6.03 a A	5.12 a AB	6.01 ab A	6.02 b B	5.25 b AB
	150 mM NaCl	*Ca*Las infection	3.73 b BC	4.49 b ABC	5.02 a ABC	5.22 a AB	4.77 b ABC	5.91 b B	3.01 c C
Starch content (µg·mm^−2^)	Control	Control	1.05 c A	1.19 c A	1.25 d A	1.33 d A	1.26 c A	1.23 d A	1.33 d A
	150 mM NaCl	Control	7.41 b A	6.53 b A	6.70 c A	6.53 c A	7.30 b A	6.67 c A	6.27 c A
	Control	*Ca*Las infection	7.51 b A	8.48 b A	8.10 b A	7.83 b A	7.97 b A	8.17 b A	8.43 b A
	150 mM NaCl	*Ca*Las infection	16.52 a B	16.98 a B	17.28 a AB	16.89 a B	17.27 a AB	17.67 a AB	18.28 a A

* Mean separation between rootstocks (columns) at a particular treatment (row) is indicated by differing uppercase letters; mean separation between treatments (rows) in a particular rootstock (column) is indicated by differing lowercase letters by Tukey’s honestly significant difference test (*p* ≤ 0.05). Val: ‘Valencia’ sweet orange, T Chl: Total chlorophyll content, NaCl: Sodium chloride.

**Table 3:** NaCl treatment and *Ca*Las infection on TPC, proline, MDA content of ‘Valencia’ sweet orange scions grafted onto S10-derived rootstocks and Cleopatra mandarin (control).

Variables	NaCl Treatment	*Ca*Las Infection	Scion/Rootstocks						
			Val/S10-1	Val/S10-2	Val/S10-3	Val/S10-4	Val/S10-5	Val/S10-6 (S10-Control)	Val/Cleo
TPC (mg gallic acid g^−1^ FW)	Control	Control	62.33 b A *	60.37 b A	60.00 ab AB	53.00 b AB	49.33 c B	60.00 b AB	57.00 b AB
	150 mM NaCl	Control	41.00 c B	45.00 c AB	50.67 b A	47.66 b AB	51.33 bc A	50.00 b A	53.00 b A
	Control	*Ca*Las infection	58.67 b B	61.33 b B	60.00 ab B	71.00 a A	60.33 b B	59.67 b B	61.00 b B
	150 mM NaCl	*Ca*Las infection	72.00 a CD	73.67 a C	66.67 a D	72.66 a C	74.67 a C	90.33 a A	80.33 a A
Proline (µmol g^−1^ FW)	Control	Control	46.00 c D	52.67 c BC	52.33 c C	53.33 c ABC	56.67 d AB	53.67 a ABC	57.00 c A
	150 mM NaCl	Control	201.13a AB	199.33 a AB	221.33 a A	174.33 a ABC	157.00 a BC	202.67a AB	118.67 ab C
	Control	*Ca*Las infection	122.33 b A	110.33 b B	115.33 b AB	114.67 b AB	109.00 c B	114.67c AB	91.33 bc C
	150 mM NaCl	*Ca*Las infection	227.00 a A	113.67 b C	121.67 b BC	128.00 b BC	141.66 b BC	158.00b B	142.00 a BC
MDA in leaves (nmol^−1^ MDA eq. g FW)	Control	Control	31.01 c B	42.75 b A	33.08 b B	33.08 d B	33.55 d B	31.48 d B	33.08 c B
	150 mM NaCl	Control	61.41 b A	38.21 b B	63.14 a A	56.28 c A	57.88 c A	49.48 c AB	63.21 b A
	Control	*Ca*Las infection	63.65 b B	88.49 a A	77.55 a AB	66.62 b AB	71.28 b AB	73.62 b AB	86.35 a AB
	150 mM NaCl	*Ca*Las infection	90.71 a ABC	86.71 a ABC	84.24 a BC	76.51 a C	93.77 a AB	90.78 a AB	96.51 a A
MDA in roots (nmol^−1^ MDA eq. g FW)	Control	Control	16.67 a A	9.67 a BC	7.27 c C	7.27 b C	7.20 c C	7.80 b BC	12.60 c AB
	150 mM NaCl	Control	19.43 a B	18.04 a B	15.37 ab B	31.5 a A	16.63 b B	23.97 a AB	18.23 b B
	Control	*Ca*Las infection	11.41 a C	12.54 a BC	12.47 bc BC	13.61b ABC	14.07 b AB	13.74 b AB	15.74 bc A
	150 mM NaCl	*Ca*Las infection	11.57 a C	11.17 a C	20.49 a BC	30.89 a AB	34.49 a A	30.89 a AB	33.89 a A

* Mean separation between rootstocks (columns) at a particular treatment (row) is indicated by differing uppercase letters; mean separation between treatments (rows) in a particular rootstock (column) is indicated by differing lowercase letters by Tukey’s honestly significant difference test (*p* ≤ 0.05). TPC: Total phenolic compounds, MDA: Lipid peroxidation (malondialdehyde equivalents).

**Table 4:** Effect of NaCl treatment and *Ca*Las infection on sodium and chloride content in leaves of ‘Valencia’ sweet orange scions and roots of S10-derived rootstocks and Cleopatra mandarin (control).

Variables	NaCl Treatment	*Ca*Las Infection	Scion/Rootstocks						
			Val/S10-1	Val/S10-2	Val/S10-3	Val/S10-4	Val/S10-5	Val/S10-6 (S10-Control)	Val/Cleo
Na(L) (mM·L^−1^)	Control	Control	37.57 c A *	35.34 c A	33.52 c A	29.66 b A	28.43 c A	28.43 c A	27.83 b A
	150 mM NaCl	Control	184.37 a AB	216.57 a A	159.74 a ABC	135.14 a BC	160.68 a ABC	94.43 b C	159.80 a ABC
	Control	*Ca*Las infection	34.60 c A	34.60 c A	36.84 c A	36.84 b A	36.93 b A	36.84 c A	37.65 b A
	150 mM NaCl	*Ca*Las infection	131.81 b AB	81.17 b B	80.19 b B	105.73 a AB	156.25 a A	152.10 a AB	174.58 a A
CL(L) (mM·L^−1^)	Control	Control	9.71 c A	8.76 b A	8.76 b A	8.76 b A	8.76 c A	8.76 b A	8.76 c A
	150 mM NaCl	Control	23.71 b AB	28.57 a AB	40.00 a A	34.47 a AB	29.05 a AB	29.71a AB	38.09 a AB
	Control	*Ca*Las infection	9.71 c A	10.00 b A	9.62 b A	9.81 b AB	8.47 c B	9.24 b AB	9.24 c AB
	150 mM NaCl	*Ca*Las infection	38.09 a A	39.43 a A	34.86 a A	34.00 a A	18.19 b B	24.29 a B	19.52 b B
Variables	NaCl treatment	*Ca*Las infection	Rootstocks						
			S10-1	S10-2	S10-3	S10-4	S10-5	S10-control	Cleo
Na(R) (mM·L^−1^)	Control	Control	73.92 c A *	74.10 b A	70.10 b AB	71.46 c AB	57.42 b AB	64.03 b AB	51.70 c B
	150 mM NaCl	Control	195.44 b C	263.22 c AB	367.46 a A	363.43 a A	235.22 a BC	325.00 a AB	232.06 b BC
	Control	*Ca*Las infection	31.95 c A	27.45 c A	29.45 b A	32.09 c A	32.46 b A	32.46 b A	27.22 c A
	150 mM NaCl	*Ca*Las infection	239.66 a B	236.53 a B	230.86 a B	250.48 a B	291.15 a AB	272.59 a AB	319.70 a A
CL(R) (mM·L^−1^)	Control	Control	6.44 b A	6.50 b A	6.53 c A	6.48 c A	6.49 b A	6.51 b A	6.48 b B
	150 mM NaCl	Control	23.71 a A	22.95 a A	23.24 a A	20.86 a A	24.00 a A	21.71 a A	25.52 a A
	Control	*Ca*Las infection	7.05 b A	7.14 b A	6.29 c B	6.00 c B	6.00 b B	6.29 b B	5.81 b B
	150 mM NaCl	*Ca*Las infection	22.95 a A	23.14 a A	16.86 b B	13.05 b C	21.90 a A	22.86 a A	18.00 b A

* Mean separation between rootstocks (columns) at a particular treatment (row) is indicated by differing uppercase letters; mean separation between treatments (rows) in a particular rootstock (column) is indicated by differing lowercase letters by Tukey’s honestly significant difference test (*p* ≤ 0.05). Na (L): Sodium content in leaves and Cl (L): Chloride content in leaves, Na (R): Sodium content in roots and Cl (R): Chloride content in roots. The sodium and chloride concentrations were expressed as mM·L^−1^.

**Table 5:** Titer values of *Ca*Las detected in leaves of ‘Valencia’ sweet orange before and after NaCl treatments.

Scion/Rootstock	Ct Value before NaCl Treatment	Ct Value after NaCl Treatment	
		0 mM NaCl	150 mM NaCl
Val/S10—line 1	28.71± 2.65 ^ab^ *	28.63 ± 0.43 ^ab^	29.86 ± 0.15 ^ab^
Val/S10—line 2	25.30 ± 0.78 ^bc^	25.26 ± 0.08 ^bc^	25.48 ± 0.33 ^bc^
Val/S10—line 3	27.77 ± 1.12 ^ab^	27.76 ± 0.03 ^ab^	27.88 ± 1.84 ^ab^
Val/S10—line 4	30.46 ± 0.30 ^ab^	29.76 ± 0.21 ^ab^	28.48 ± 3.35 ^ab^
Val/S10—line 5	31.45 ± 1.22 ^a^	30.15 ± 0.23 ^a^	28.27 ± 3.01 ^ab^
Val/S10—line 6 (S10-control)	27.72 ± 0.64 ^ab^	27.02 ± 0.25 ^b^	24.59 ± 1.42 ^bc^
Val/Cleo	26.87 ± 0.61 ^ab^	25.4 ± 0.55 ^e^	19.50 ± 0.99 ^c^

* Means separation by Tukey’s honestly significant difference test (*p* ≤ 0.05).

## Data Availability

Data are contained within the article.

## Supplementary Materials

The following are available online at https://www.mdpi.com/article/10.3390/plants10071439/s1, Table S1: Significance analysis of physiological traits of ‘Valencia’ scions grafted onto S10 rootstocks and cleopatra mandarin using a three-way ANOVA assay. Table S2. List of the primer sequences used in real-time PCR assay.
