# Supplementary material for: Physiological Responses and Gene Expression Patterns in Open-Pollinated Seedlings of a Pummelo-Mandarin Hybrid Rootstock Exposed to Salt Stress and Huanglongbing
Source: Plants (Basel). 2021 Jul 14;10(7):1439. doi: 10.3390/plants10071439 (PMC8309399; doi:10.3390/plants10071439)
Supplement: Supplementary file 1 [file plants-10-01439-s001.zip › plants-1278490-supplementary.pdf]

Supplemental Table S1. Significance Analysis of physiological traits of 'Valencia' scions grafted onto S10 rootstocks and cleopatra mandarin using a three-way ANOVA assay.

Variables	Effect	DF	*F*-value	*p*-value	CV
T Chl	Rootstocks	6	10.97	**<.0001**[a]	
	NaCl treatments	1	55.53	**<.0001**	
	CaLas infection	1	96.79	**<.0001**	
	Rootstocks*NaCl treatments	6	2.44	0.0359	
	Rootstocks**CaLas* infection	6	3.56	**0.0047**	
	NaCl treatments**CaLas* infection	1	1.28	0.2616	
	Rootstocks*NaCl treatments**CaLas* infection	6	2.13	0.0636	
	Total	27	9.93	**<.0001**	11%
Starch	Rootstocks	6	0.81	0.5649	
	NaCl treatments	1	2838.79	**<.0001**	
	CaLas infection	1	3926.62	**<.0001**	
	Rootstocks*NaCl treatments	6	0.57	0.7517	
	Rootstocks**CaLas* infection	6	2.33	**0.0444**	
	NaCl treatments**CaLas* infection	1	175.49	**<.0001**	
	Rootstocks*NaCl treatments**CaLas* infection	6	1	0.4318	
	Total	27	258.11	**<.0001**	7%
MDA (leaves)	Rootstocks	6	2.9	**0.0006**	
	NaCl treatments	1	96.85	**<.0001**	
	CaLas infection	1	471	**<.0001**	
	Rootstocks*NaCl treatments	6	8.06	**<.0001**	
	Rootstocks**CaLas* infection	6	1.9	**0.0004**	
	NaCl treatments**CaLas* infection	1	10.18	**0.0012**	
	Rootstocks*NaCl treatments**CaLas* infection	6	1.043	**0.0382**	
	Total	27	44.94	**<.0001**	9%
MDA (roots)	Rootstocks	6	13.15	**<.0001**	
	NaCl treatments	1	273.03	**<.0001**	
	CaLas infection	1	35.03	**<.0001**	
	Rootstocks*NaCl treatments	6	15.97	**<.0001**	
	Rootstocks**CaLas* infection	6	13.61	**<.0001**	
	NaCl treatments**CaLas* infection	1	0.3	0.586	
	Rootstocks*NaCl treatments**CaLas* infection	6	5.66	**<.0001**	

	Total	27	22.17	**<.0001**	17%
TPC	Rootstocks	6	4.2482	**0.0014**	
	NaCl treatments	1	8.3458	**0.0055**	
	CaLas infection	1	335.29	**0.0032**	
	Rootstocks*NaCl treatments	6	6.75	**<.0001**	
	Rootstocks**CaLas* infection	6	3.77	**<.0001**	
	NaCl treatments**CaLas* infection	1	178.47	**<.0001**	
	Rootstocks*NaCl treatments**CaLas* infection	6	7.08	**<.0001**	
	Total	27	24.19	**<.0001**	6%
Proline	Rootstocks	6	14.63	**<.0001**	
	NaCl treatments	1	799.95	**<.0001**	
	CaLas infection	1	15.97	**0.0002**	
	Rootstocks*NaCl treatments	6	10.31	**<.0001**	
	Rootstocks**CaLas* infection	6	9.71	**<.0001**	
	NaCl treatments**CaLas* infection	1	251.23	**<.0001**	
	Rootstocks*NaCl treatments**CaLas* infection	6	11.8	**<.0001**	
	Total	27	49.85	**<.0001**	10%
Na (L)	Rootstocks	6	3.75	**0.0033**	
	NaCl treatments	1	731.34	**<.0001**	
	CaLas infection	1	12.13	**0.001**	
	Rootstocks*NaCl treatments	6	3.73	**0.0034**	
	Rootstocks**CaLas* infection	6	10.17	**<.0001**	
	NaCl treatments**CaLas* infection	1	21.86	**<.0001**	
	Rootstocks*NaCl treatments**CaLas* infection	6	7.99	**<.0001**	
	Total	27	34.04	**<.0001**	20%
Na (R)	Rootstocks	6	2.7	**0.0224**	
	NaCl treatments	1	950.42	**<.0001**	
	CaLas infection	1	14.64	**0.0003**	
	Rootstocks*NaCl treatments	6	2.98	**0.0134**	
	Rootstocks**CaLas* infection	6	5.62	**0.0001**	
	NaCl treatments**CaLas* infection	1	1.14	0.2902	
	Rootstocks*NaCl treatments* *CaLas* infection	6	4.51	**0.0008**	

	Total	27	20.35	**<.0001**	23%
Cl (L)	Rootstocks	6	3.42	**0.006**	
	NaCl treatments	1	453.59	**<.0001**	
	CaLas infection	1	0.64	0.4261	
	Rootstocks*NaCl treatments	6	2.85	**0.017**	
	Rootstocks**CaLas* infection	6	4.76	**0.0006**	
	NaCl treatments**CaLas* infection	1	1.78	0.1869	
	Rootstocks*NaCl treatments**CaLas* infection	6	4.56	**0.0008**	
	Total	27	20.35	**<.0001**	23%
Cl (R)	Rootstocks	6	3.82	**0.0029**	
	NaCl treatments	1	1034.78	**<.0001**	
	CaLas infection	1	13.4	**0.0006**	
	Rootstocks*NaCl treatments	6	3.06	**0.0114**	
	Rootstocks**CaLas* infection	6	2.84	**0.0171**	
	NaCl treatments**CaLas* infection	1	11.75	**0.0011**	
	Rootstocks*NaCl treatments**CaLas* infection	6	1.99	0.0821	
	Total	27	41.86	**<.0001**	15%

[a]Bold value indicates a significant difference. Df: degree of freedom, CV: coefficient of variation, NaCl: sodium chloride, T Chl: total chlorophyll content, TPC: total phenolic compounds, MDA: lipid peroxidation (malondialdehyde equivalents), Na (L): sodium content in leaves and Cl (R): chloride content in leaves, Na (R): sodium content in roots and Cl (R): chloride content in roots.

Supplemental Table S2. List of the Primer sequences used in real-time PCR assay

NO.	Gene	Common name	Group	Forward Seq	Reverse Seq
1	orange1.1g026287m	CsCSD2	Antioxidant enzymes	CGCTCTTCCTCTTCTTCTTCTT	CGGCGAGAGATAAGTTGAGAC
2	orange1.1g005031m	CsPAL1		CTCGATGGCAGCTCTTATGTTA	GGTGAAGTTCTCAGGGCATAA
3	orange1.1g001116m	CsSOS1	Na+ co-transporter	GCCAAGTGGTATCTGGCTTAT	GCACCTCATAGAGACCCAAATTA
4	orange1.1g013421m	CsSOS2		GCGAGGAAGAGGAAGTGAAT	GAGAGGACCTCCGACTTTATTT
5	orange1.1g027657m	CsSOS3		TTCGATCATTGGGTGTCTTCC	AACTCCTCCCGCTCAATAAAC
6	orange1.1g009116m	CsNHX1		GAGCTTTGACCTCTCTCACATC	GCACTAAGCAGTCCAGCTATAA
7	orange1.1g048073m	CsPR1	Pathogen related proteins	GTGGCGGAGAAAGCTAACTATAA	AACCCTAGCACATCCAACAC
8	orange1.1g019014m	CsPR2		ACAACCCAGTACGTGTCTTTC	TGCCGTGGAAACTTTGATTTG
9	orange1.1g017124m	β-actin	Housekeeping	GCTGCCTGATGGCCAGATC	AGTTGTAGGTAGTCTCATGAA
